# The Effect of Hokkaido Red Wines on Vascular Outcomes in Healthy Adult Men: A Pilot Study

**DOI:** 10.3390/nu15184054

**Published:** 2023-09-19

**Authors:** Prae Charoenwoodhipong, Roberta R. Holt, Carl L. Keen, Nasim Hedayati, Tomoyuki Sato, Teruo Sone, Robert M. Hackman

**Affiliations:** 1 Department of Nutrition, University of California Davis, Davis, CA 95616, USA; pcharoen@ucdavis.edu (P.C.);; 2 Department of Internal Medicine, University of California Davis, Sacramento, CA 95817, USA; 3 Division of Vascular Surgery, Department of Surgery, University of California Davis, Sacramento, CA 95817, USA; 4 Research Faculty of Agriculture, Hokkaido University, Sapporo 060-8589, Hokkaido, Japan

**Keywords:** red wine, vascular function, blood pressure, anthocyanins, polyphenols, arterial stiffness, augmentation index, hydroxytyrosol

## Abstract

Moderate red wine intake has been associated with lower cardiovascular mortality, due in part to the intake of polyphenols and anthocyanins, whose content can vary from varietal and year of harvest. This study assessed the vascular effects in response to a single intake of 2015 and 2018 Zweigelt red wines from Hokkaido, Japan. Healthy men were randomly assigned to consume 240 mL each of a red wine, or a sparkling white grape juice as a control in a randomized three-arm cross-over design with a 7 day washout between arms. The augmentation index (AI; a measure of arterial stiffness) and AI at 75 beats/min (AI75), reactive hyperemia index, systolic and diastolic blood pressure (SBP and DBP, respectively), and platelet reactivity were assessed at baseline and two and four hours after each beverage intake. Changes from the baseline were analyzed using a linear mixed model. Significant treatment effects (*p* = 0.02) were observed, with AI 13% lower after the intake of the 2015 or 2018 vintages compared to the control. Intake of the 2018 vintage reduced SBP and DBP (−4.1 mmHg and −5.6 mmHg, respectively; *p* = 0.02) compared to the 2015 wine and the control drink. The amount of hydroxytyrosol in the 2018 wine was almost twice the amount as in the 2015 wine, which may help explain the variable blood pressure results. Future studies exploring the vascular effects of the same red wine from different vintage years and different phenolic profiles are warranted.

## 1. Introduction

Moderate intake of red wine has been associated with beneficial effects on cardiovascular health [1,2]. The bioactivity of red wine is thought to be due in part to the intake of polyphenols [3,4]. Anthocyanins, a group of polyphenols known for their beneficial effects on cardiovascular health, strongly influence red wine color and hue [5,6,7]. Higher anthocyanin intake has been associated with reduced arterial stiffness and blood pressure in women aged 18–75 years [8]. However, major red wine anthocyanins, such as malvidin glucoside (MG), can vary between the year of production, be higher in younger wines, and is affected by vintage years [9,10,11]. The polyphenolic content of wine can also be influenced by environmental factors such as temperature, humidity, light exposure, and soil and growing conditions [12,13]. Similarly, yearly variations in yeast biodiversity may contribute to differences in the polyphenolic profiles for different red wine vintages [14,15]. Changes in polyphenolic characteristics such as hue and color intensity have been noted in the same wine produced in different vintage years [16,17,18]. Differences in anthocyanin concentration and the color appearance were observed in the same cultivars planted in two areas in China with contrasting geographies and climates [19].

**Figure 1:**
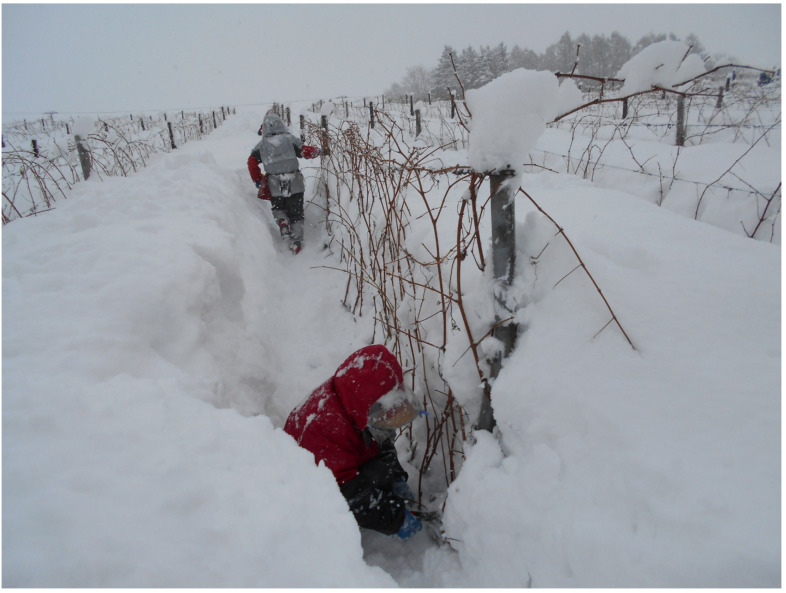
Winter vineyard pruning in Hokkaido, Japan (photo used with permission from Hokkaido Wine Co.; Otaru, Hokkaido, Japan).

Given the above, the polyphenolic profile can vary substantially between red wines produced from the same grape varietal in different years and location [20,21]. Whether the variation in anthocyanin content, along with other polyphenols, can result in different cardiovascular health outcomes is unknown. Therefore, this study explored the effects of a single intake of the same red wine varietal produced in a similar geographic area, but from different vintage years, on indices of vascular function, blood pressure, and platelet aggregation in healthy adult men. A Zweigelt red wine grape varietal grown in Hokkaido, Japan, was selected. In Hokkaido, vineyards are typically covered with heavy snow during the winter (Figure 1), and grape production occurs during a short summer season with fewer hours of daily sunlight compared to varietals from warmer climates and longer days of summer sunlight that produce the majority of the world’s wine [22].

## 2. Materials and Methods

### 2.1. Recruitment

Healthy men aged 50 to 70 years were recruited through flyers, newspapers, and online resources at the University of California, Davis (UC Davis). Criteria for inclusion were a body mass index (BMI) of 18.5–40 kg/m^2^, body weight ≥ 110 pounds (49.9 kg), self-reported stable dose of prescription medication for the past six months (if taking any), non-smoker, and regular consumer of alcoholic beverages (between two drinks/week to two drinks/day). One standard drink of alcoholic beverage was defined as 355 mL (12 oz.) of beer (5% alcohol), 237 mL (8 oz.) of malt liquor (7% alcohol), 148 mL (5 oz.) of wine (12% alcohol), or 1.5 oz. of 80-proof distilled spirits or liquor (40+% alcohol). Exclusion criteria were daily use of aspirin or non-steroidal anti-inflammatory drugs, dislike of wine, grapes, or alcohol, following a non-traditional diet (e.g., vegan or vegetarian), fruit consumption ≥ 364 gm (2 cups)/day, vegetable intake ≥ 546 gm (3 cups)/day, consuming fatty fish or coffee/tea ≥ three portions/week, or eating dark chocolate ≥ 85 gm (3 oz.)/day. Self-reported restriction of physical activity or chronic/routine high-intensity exercise were also exclusions, as were blood pressure ≥ 140/90 mm Hg, disorders that could affect vascular function (e.g., diabetes mellitus, renal or liver diseases, and cardiovascular events or stroke), or indications of substance or alcohol abuse. Volunteers were asked to refrain from using multivitamin and mineral supplements other than a general formula that met up to 100% of the United States recommended dietary allowance, and if applicable, were required to discontinue the intake of supplements containing botanical ingredients or fish oil for at least a month before study enrollment. Abnormal values from a comprehensive metabolic panel (CMP) and complete blood count (CBC) were exclusions unless approved by the study physician. The University of California Davis Medical Center’s Department of Pathology and Laboratory Medicine performed the CMP and CBC analyses. The Institutional Review Board of the University of California, Davis approved the study protocol, with the study registered on ClinicalTrials.gov: NCT05138939.

### 2.2. Study Design and Procedures

Those eligible for enrollment were randomized into a three-arm, controlled cross-over study. Twenty-four hours prior to the study day, participants were instructed to refrain from soda, sports drinks, flavored water, and polyphenol-rich food, particularly olives, berries, apples, beans, citrus, onions, nuts, herbs, coffee, tea, beer, wine, cocoa, and chocolate products or beverages as these foods might confound the outcomes. Participants fasted for at least 12 h, with the measurements of vascular function, blood pressure, and blood sampling performed at baseline and two and four hours after beverage consumption. After baseline measurements, the participants were provided in a single-blinded fashion 240 mL (8 oz.) of one of the two Zweigelt red wines from the 2015 or the 2018 vintages or a sparkling white grape juice (Welch’s, Concord, MA, USA) as a control. The beverages were provided along with a small snack consisting of low-moisture part-skim mozzarella string cheese (Galbani-Dal 1882, USA; 160 kcals, 12 g fat, 14 g protein, and 0 g carbohydrate) and 16 crackers (200 kcal; Carr’s table water crackers, UK; 4 g fat, 4 g protein, and 40 g carbohydrate). Hokkaido Wine Co., Ltd. produced the wines from Zweigelt grapes (also known as Rotburger [23]) grown in Hokkaido, Japan. The wine was dispensed using a system that placed a probe through a cork in the neck of the wine bottle, and after each pouring of wine, argon gas was injected into the headspace in order to preserve freshness and chemical composition (Coravin, Bedford, MA, USA). The nutritional composition of the sparkling white grape juice was 110 kcal containing 28 g of total sugar, with an additional 24 g (80 kcal) of granulated sugar added by the investigators in order to match the caloric content of the red wines, which was approximately 190 kcal per serving [24].

### 2.3. Chemical Composition and Polyphenolic Profiles

The basic chemical characteristics of the wines were provided by the manufacturer. Independent analyses of the polyphenolics (ETS Laboratories, St. Helena, CA, USA) for the two red wines were conducted prior to the planned start of the intervention in 2019 and again at trial completion in 2022, which also included the sparkling white grape juice control. In 2022, the total polyphenol content (TPC in mg gallic acid equivalents (mg GAE)) of all three beverages was measured in triplicate according to the manufacturer’s instructions (Zen-Bio, Durham, NC, USA). Briefly, the beverages were diluted in water at a ratio of 1:10, then 10 µL of the diluted samples was incubated with a 10% Folin–Ciocalteu reagent for two hours and absorbance was measured at 765 nm using a Synergy H1 plate reader (BioTek, Winooski, VT, USA).

Tyrosol (Tyr) and hydroxytyrosol (HT) concentrations were measured from each of the two Hokkaido red wines. Briefly, the wine was filtered through a 0.45 µM nylon filter and then analyzed in duplicate using a direct liquid chromatography method with a diode array detector as previously described [25,26].

### 2.4. Assessment of Vascular Function

Prior to the vascular function measurement, participants rested in a seated position for 15 min, after which systolic blood pressure (SBP), diastolic blood pressure (DBP), and heart rate were measured three times, five minutes apart, with a digital blood pressure device (Welch Allyn, NY, USA). Data were calculated as the average of three readings.

Peripheral arterial tonometry (PAT; Endo-PAT2000; Itamar Medical, Israel) was used to monitor changes in digital pulsatile arterial volume [27]. Participants rested in a supine position for 10 min prior to a supine blood pressure measurement required for the system settings. After baseline collection for six minutes, a five-minute occlusion was performed by inflating a lower-arm blood pressure cuff 60 mmHg above the supine SBP. Reactive hyperemia is the phenomenon of reperfusion of blood to the ischemic area following pressure cuff release [28,29]. The system software automatically calculated the reactive hyperemia index (RHI) as the ratio of the average pulse wave amplitude (PWA) during a one-minute period following 90 s of reactive hyperemia to the average PWA during a three-to-five-minute baseline period, with the same ratio in the non-occluded arm serving as a control [30]. The RHI measures peripheral microvascular function in the digital vasculature that reflects, in part, nitric oxide-dependent vasodilation [31,32,33]. Relationships between the RHI response and circulating nitrate and epoxyeicosatrienoic acid levels have been reported [34,35]. The natural logarithmic transformation of the RHI ratio during data collection from 90 to 120 s after the release of the occlusion was calculated along with the Framingham reactive hyperemia index (fRHI), which has been correlated with cardiovascular risk factors [36]. An RHI value of less than 1.67 has been correlated with endothelial dysfunction and an index higher than this number represents normal endothelial function [27].

The Augmentation Index (AI), a measure of peripheral arterial stiffness, was calculated from the baseline PAT waveform as the difference between the first (P1) and second (P2) peaks of the central arterial waveform (i.e., [P2 − P1]/P1 × 100%). The AI was also standardized to a heart rate of 75 beats per minute (AI75). A lower AI value represents greater elasticity in the arteries (i.e., less stiffness).

### 2.5. Platelet Aggregometry

Optical platelet aggregometry was performed in citrated blood using a two-channel Chrono-Log 700 device (Havertown, PA, USA). Fifteen minutes after blood collection, platelet-rich plasma (PRP) was separated from whole blood by centrifugation (200× *g* for 10 min at 25 °C). The upper 75% of the PRP layer was aliquoted into a separate tube, and then platelet-poor plasma (PPP) was obtained by further centrifugation of the whole blood tubes at 1500× *g* for 10 min at 25 °C. After resting the PRP for a minimum of 15 min, the platelet aggregation testing commenced. Aliquots of 500 µL of PRP were incubated at 37 °C for a minimum of three minutes prior to stimulation with three agonists: collagen at a concentration of either one or three µg/mL or 10 µM adenosine diphosphate (ADP). The aggregation tests were performed in duplicate at a stirring speed of 1200 rpm and showed intra-assay mean and standard error of 10 ± 2%. After 10 min of data collection, the software generated values for area-under-the-curve (AUC), maximal aggregation (maxA), and slope from the response of activated samples.

### 2.6. Twenty-Four-Hour Dietary Recall

Dietary intake data for 24-h recalls were collected and analyzed using the Automated Self-Administered 24-h (ASA24) Dietary Assessment Tool, version (2020), developed by the National Cancer Institute, Bethesda, MD, USA. A recall was taken during each study visit, representing the days participants were asked to restrict polyphenol-rich foods, to check for compliance. The other two dietary recalls were completed by the participants at their convenience, which represented their usual intake.

### 2.7. Statistical Analyses

A linear mixed model was used to assess changes from baseline in vascular outcomes, blood pressure, and range-scaled platelet function using time and intervention groups as the main effects and individual participants as the random effect (JMP, version 16; Cary, NC, USA). Post hoc analyses were conducted using significant effects of time, treatment, or their interactions with Tukey’s test. A one-way ANOVA assessed reported differences in food intake using the intervention group as the main factor. Similarly, a one-way ANOVA was used to assess differences in baseline values of each parameter among different interventions. Data not normally distributed were adjusted using Johnson’s transformation prior to analysis. Unless indicated otherwise, data are reported as mean ± standard deviation (SD). The least-squares mean (LSM) of the observed values that showed significant differences between intervention groups was used to illustrate the data in bar graphs.

## 3. Results

### 3.1. Chemical Composition and Polyphenolic Profiles

Table 1 presents the basic chemical characteristics of the two red wines. Both wines had similar specific gravity, alcohol content, acidity, and pH, with the Zweigelt 2015 34% lower in total sulfur dioxide (SO_2_), the sum of molecular, free, and bound SO_2_ (2015: 98 and 2018: 149 ppm) and 38% higher in sugar (2015: 5.6 and 2018: 3.5 g/L) than Zweigelt 2018.

Table 2 presents the polyphenolic profiles from an initial analysis conducted in 2019 and a subsequent analysis conducted in 2022 following a 17-month clinical laboratory closure due to the COVID-19 pandemic. While the 2018 Zweigelt wine was substantially higher in total anthocyanin content in 2019, it declined 76% by 2022. A decrease in monomeric anthocyanin content primarily influenced this observation, which was approximately 64 mg in 2019 but had reduced to 8.4 mg in 2022. Even with these reductions, the total and monomeric anthocyanin content for the 2018 wine was still 34 and 45%, respectively, higher than the 2015 Zweigelt, with the later wine showing a decrease in total anthocyanin and monomeric anthocyanin content by 49% and 71%, respectively. In contrast, the TPC content of the 2015 Zweigelt was 16% greater in 2022 compared to the 2018 wine (674 vs. 583 mg GAE, respectively), with both wines having a substantially higher TPC content compared to the sparkling white grape juice (104 mg GAE).

The amount of Tyr and HT in the 2015 and 2018 Zweigelt red wines is shown in Table 3. The amount of HT was almost twice as large in the 2018 Zweigelt red wine compared to the 2015 vintage, while the amount of Tyr was approximately 45% greater in the 2015 wine compared to its 2018 counterpart.

### 3.2. Demographics and Baseline Characteristics

**Figure 2:**
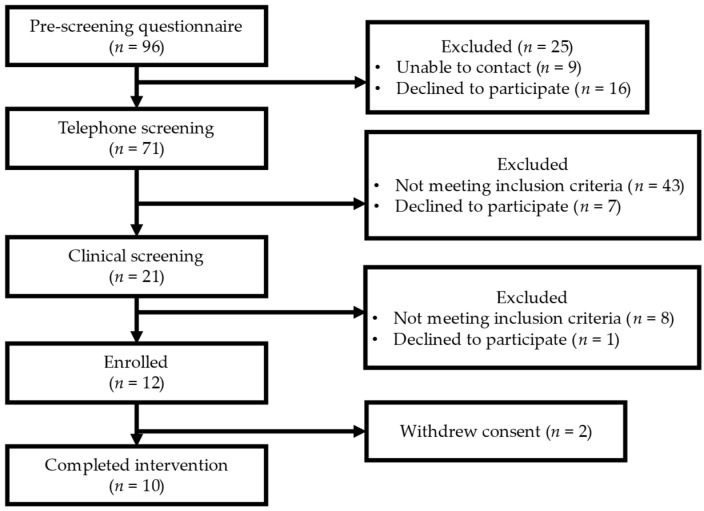
Recruitment and enrollment.

Ten men completed the study, which spanned from September 2021 to June 2022 (Figure 2). Their baseline demographic, glucose, platelet count, and vascular function characteristics are shown in Table 4. Their baseline dietary characteristics are shown in Table S1. Eight participants self-reported their race as Caucasian or White, one as African-American or Black with European ethnicity (French, Spanish, and Greece), and one self-reported as Spanish-American or Latino. On average, the participants were 58.6 years of age, in the overweight range for BMI, with their CBC and CMP values within the normal reference ranges. At baseline, participants’ glucose levels and platelet counts were within the normal range of 98.60 ± 10.81 mg/dL and 234.05 ± 87.60 K/MM^3^, respectively. On average, the participants were considered pre-hypertensive with a SBP of 124.47 ± 2.70 mmHg, while DBP and heart rate (HR) were 82.20 ± 1.23 mmHg and 63.5 ± 2.68 bpm, respectively (mean ± SEM). The AI and AI75 values were 17.62 ± 6.46 (% pulse pressure) and 7.70 ± 5.99 (% pulse pressure), respectively, with a normal RHI and fRHI at 2.25 ± 0.10 and 0.78 ± 0.11 (mean ± SEM).

### 3.3. Arterial Stiffness

**Figure 3:**
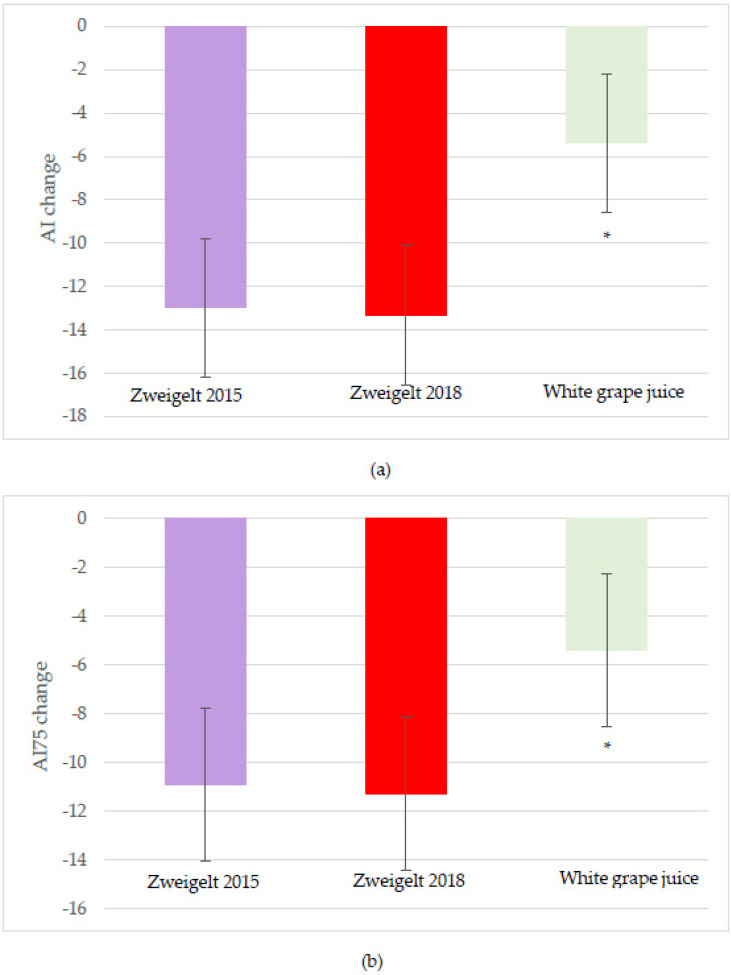
Least-squares mean (LSM) plot of the intervention group effects for the overall change from baseline for (**a**) augmentation index; and (**b**) augmentation index adjusted to 75 bpm. A linear mixed model was used to assess changes from baseline using time and intervention groups as the main effects and individual participants as the random effect. Data are the LSM ± SEM; * Significantly different at the *p* < 0.05 level after Tukey’s post hoc testing. AI, Augmentation Index; AI75, Augmentation Index adjusted to 75 bpm; bpm, beats per minute.

The changes from baseline at two and four hours after beverage intake for vascular outcomes, including the AI and AI75 values (measures of arterial stiffness), are shown in Table 5. No significant interactive effects were observed. A significant effect for the red wine (0.002) was observed, with the overall change in AI lower after the intake of the 2015 and 2018 vintages compared to the control values (−12.98 ± 3.20% and −13.34 ± 3.20%, respectively, vs. control −5.38 ± 3.20%; *p* < 0.05; Figure 3a). Significant time effects included a lower two-hour change in AI with the intake of the 2018 vintage (−18.27 ± 3.58%) compared to the control (−7.97 ± 2.67%; *p* < 0.05). Similarly, significant time (*p* = 0.01) and intervention group (*p* = 0.04) effects for AI75 were noted, with intervention group trends for lower AI75 with the intake of the Zweigelt 2015 (−10.92 ± 3.13; *p* = 0.09) and 2018 (−11.28 ± 3.13; *p* = 0.06) wines compared to the sparkling white grape juice control (−5.41 ± 3.13; Figure 3b).

### 3.4. Reactive Hyperemia Index

No significant interactive or intervention group effects for RHI or fRHI were observed. A significant change (*p* = 0.003) in RHI for time was noted with the intake of the Zweigelt 2015 wine over the entire four-hour period (0.74 ± 0.24) compared to the first two hours (−0.04 ± 0.13), with similar trends observed for fRHI (Table 5).

### 3.5. Blood Pressure

**Figure 4:**
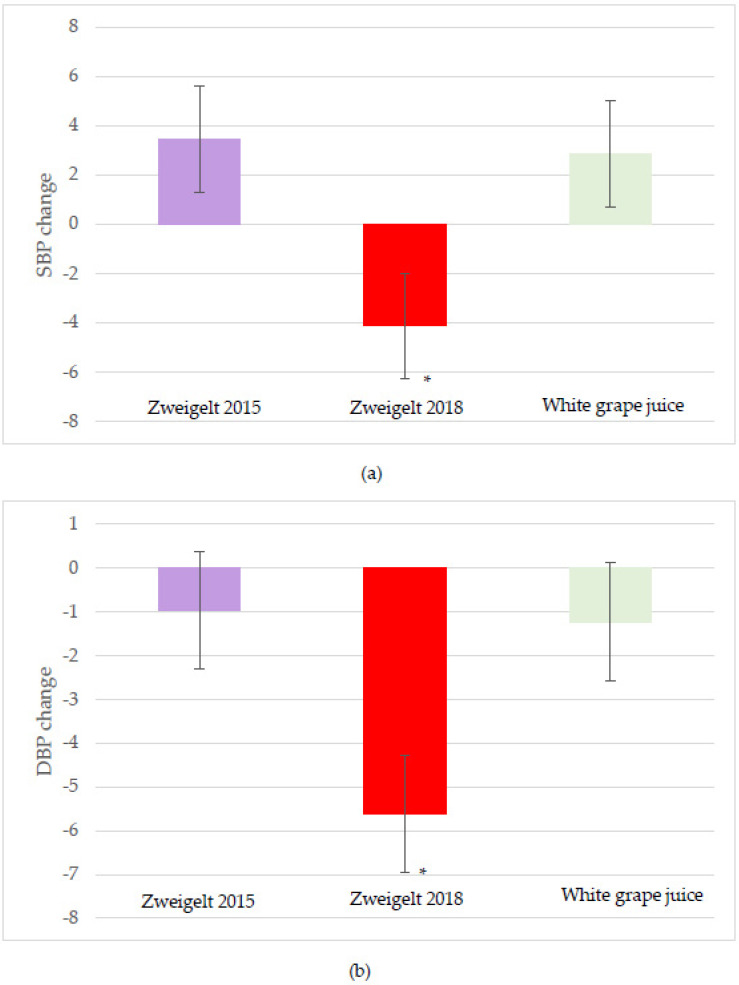
Least-squares mean (LSM) plot of the intervention group effects for the overall change from baseline for (**a**) systolic; and (**b**) diastolic blood pressure. A linear mixed model was used to assess changes from baseline using time and intervention groups as the main effects and individual participants as the random effect. Data are the LSM ± SEM; * significantly different at the *p* < 0.05 level after Tukey’s post hoc testing. SBP, systolic blood pressure; DBP, diastolic blood pressure.

**Figure 5:**
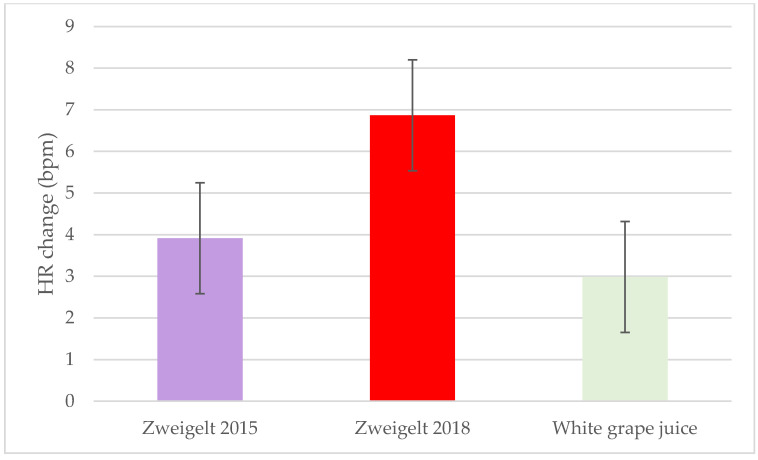
Least-squares mean plot of overall change from baseline of heart rate. A linear mixed model was used to assess changes from baseline using time and intervention groups as the main effects and individual participants as the random effect. Data are the LSM ± SEM; HR, heart rate; bpm, beats per minute.

Significant treatment effects were observed for both SBP and DBP, with the overall changes lower following intake of the 2018 vintage compared to the 2015 vintage or the control beverage (−4.1 SBP and −5.6 DBP mmHg; *p* = 0.02) (Figure 4a,b). No significant interactive or time effects for SBP and DBP were noted. A strong time effect was observed for the HR, with the two-hour change greater after consumption of the 2018 wine compared to the other groups (*p*= 0.051; Figure 5). No other significant changes in HR were observed.

### 3.6. Platelet Aggregation

Significant time effects were observed for the overall changes in MaxA, slope, and AUC following stimulation with the 3 µg/mL collagen agonist, with the two-hour change in platelet reactivity significantly higher than the four-hour change after consumption of each beverage (Supplementary Table S2). No significant interactive or intervention group effects were noted for other platelet function parameters (Supplementary Table S3).

### 3.7. Dietary Intake

No significant differences were noted in reported dietary intake between the three interventions. However, when comparing dietary intake among study visits, the consumption of fats (saturated, total, and solid fats), iron, and folate was significantly reduced in the second and third visits compared to the first study day (Supplementary Tables S4 and S5).

## 4. Discussion

The objective of this study was to assess the influence of the intake of two red wines of the same varietal and region, but from different vintage years, on vascular and platelet function. The main finding was that intake of either the 2015 or 2018 Zweigelt red wines, along with a small snack providing 360 kcal (40% of calories from fat), lowered AI compared to the intake of a sparkling white grape juice control. In addition, SBP and DBP were significantly reduced in the postprandial period with the 2018 wine compared to the 2015 wine or the control beverage.

With frequent consumption of excessive calories, fats, and refined sugars in the modern Western diet, a postprandial state that typically lasts 6–12 h can extend to more than 16 h [37,38]. The prolonged postprandial state can increase cardiovascular risk through increased exposure to elevated plasma glucose, triglyceride-rich VLDLs, chylomicrons and their remnants, which induce inflammation, oxidative stress, immune imbalances, and endothelial dysfunction to promote atherosclerosis [37,39]. Atherosclerosis, along with vascular aging, endothelial dysfunction, and structural remodeling, results in increased arterial stiffness, which can be measured by pulse wave velocity (PWV) and AI [40,41].

In the postprandial state, vascular function responses, including PWV, AI, and BP, may vary depending on food composition. Imbalanced macronutrients such as high calories, saturated fat, and simple carbohydrates can induce unfavorable postprandial responses, including inflammation, oxidative stress, and endothelial dysfunction [42,43]. In the current study, a reduction in blood pressure and AI and a trend toward increased HR following beverage and food intake are consistent with the aforementioned postprandial studies that report a decrease in AI two [28] and four hours after a high fat meal [44] or a standardized breakfast [45]. The significantly greater reduction in AI with the intake of the 2015 or 2018 red wines compared to sparkling white grape juice, all consumed with a snack, could be due to the presence of additional bioactive compounds in the meal, including alcohol and certain polyphenols such as anthocyanins that were not present in the control. Light-to-moderate alcohol consumption (15 g for women and 30 g for men) has been associated with lower arterial stiffness [40], while polyphenols in red wine, including anthocyanins, flavan-3-ols, phenolic acids, ellagitannins, and resveratrol, have been reported to have anti-inflammatory, anti-oxidative, and vasodilating properties [4,46]. The individual and interactive effects of alcohol and polyphenols may help explain the more favorable vascular response in red wine compared to other beverages. In two separate studies, the intake of red wine with a meal (a slice of white bread (30 g) and 30 g of cottage cheese (4% fat); 107 kcal, 6 g protein, 2 g fat, 15 g carbohydrate) significantly improved flow-mediated dilation (FMD) and AI [47,48]. Interestingly, the increase in FMD response was greater when red wine was combined with the intake of green olive oil [47]. A similar response was not observed with white wine intake, suggesting that the phenolic content in the red wine was a key to an improved vascular response [48]. The results from our study of an improved vascular response with red wine in contrast to a sparkling low phenolic white grape juice control are in agreement with these results.

The significant reduction in SBP and DBP following intake of the 2018 vintage compared to the 2015 red wine or the white grape juice control suggests differences in bioactive constituents between the two vintages. For the most part, the 2015 and 2018 red wines were similar in alcohol content and polyphenolic profiles (Table 2). However, the 2018 red wine contained almost twice as much HT compared to the 2015 wine, which is of interest since hydroxytyrosol has been shown to be better absorbed than Tyr in in vitro models [49], as well as more bioavailable than Tyr in human studies [50]. Hydroxytyrosol supplementation has been shown to reduce blood pressure in a diabetic rat model [51] and counteract endothelin-1 expression, a hypertensive agent [52].

Apart from red wine, HT is a major olive oil phenolic [53]. A sub-analysis of the Prevención por Dieta Mediterránea (PREDIMED) study showed a positive association between participants’ urinary HT and alcohol consumption that was primarily from red wine [54]. Additionally, metabolites of phenolic compounds found in wine, including resveratrol [55,56] and HT [54] have been associated with alcohol intake. Hydroxytyrosol can also be produced endogenously as a metabolite of tyramine from dopamine metabolism [57]. Researchers from the PREDIMED study subsequently conducted two randomized, cross-over, controlled clinical trials to understand the disposition of HT by comparing the 24 h pharmacokinetics from one study following a single intake of red wine (250 mL; 0.35 mg HT) and another study with extra virgin olive oil (EVOO: 25 mL; 1.7 mg) [57]. The results showed that urinary HT levels after red wine intake were significantly higher than those from EVOO [57], suggesting the unique properties in red wine that might promote endogenous production of HT. In another study, urinary HT concentration was assessed over a six-hour period after intake of a single serving (147 mL) of vodka, red wine, dealcoholized red wine, or water in 28 healthy male adults (average age of 26.6 years). Urinary levels were significantly greater for those consuming red wine, dealcoholized red wine, or vodka than from the water group, suggesting that alcohol and/or phenolic compounds aid in de novo HT generation [58]. Another sub-analysis from the PREDIMED study reported a significant association between the higher concentration of the HT metabolite, homovanillyl alcohol, and the lower mortality rate and less CVD burden [59]. Since HT can be absorbed and metabolized within four to six hours, as observed in the studies utilizing red wine [58] and olive oil [60], its presence in higher amounts in the 2018 Hokkaido red wine than in the 2015 vintage might help explain the discrepancies in blood pressure observed in the present study.

In addition to HT, anthocyanins, and ellagitannins, the primary polyphenols in red wines may also influence vascular outcomes. Since the current study assessed the effects of red wines on vascular outcomes over four hours, the absorption and metabolism of anthocyanins and ellagitannins would likely be minimal [61,62]. Future studies of longer duration should also consider a more precise differentiation of compounds within these two categories, since previous studies have reported favorable cardiovascular outcomes depending on the major subtypes of anthocyanins [63,64] or different ellagitannin profiles of red wine from different vintage years [65].

A strength of this study is the assessment of clinical responses from the same type of wine, vinted from the same grape cultivar, grown in a similar geographic region, but from two different years. This novel study design has not been employed previously, to our knowledge. Numerous studies have reported significant differences in the content and type of polyphenols in red grapes or red wines produced under different environmental stresses [15,20,66,67], and it is reasonable to hypothesize that these chemical differences may produce differences in vascular outcomes. Another strength is the detailed polyphenolic profiles of the red wines and sparkling white grape juice. Many previous studies provide little or no compositional information about the red wines tested [68,69,70,71,72]. Future studies should provide a detailed chemical profile of the test wine(s) to better enable interpretation of results and make more accurate comparisons between studies.

The red wine in the current study was produced in Hokkaido, Japan, where grapevines are covered by snow during the winter and experience a short, cool summer season [22]. A higher concentration of phenolic compounds has been reported in cultivars grown in climates of long winters, low temperatures, and possible snowfall [73]. However, the total polyphenolic content of the wines tested here is similar to the vast majority of studies on vascular function that have assessed wines from warmer climates with longer growing seasons [74]. Unfortunately, the influence of any particular polyphenol on vascular or other physiologic responses cannot be evaluated from most other studies, which report little detailed analysis of the test wines. Chemical profiling is also of interest when comparing the same red wine across different vintage years. Such detail is important since results from the present study demonstrate that the 2018 red wine, relatively rich in HT, produced a significant reduction in blood pressure, while the 2015 vintage (with approximately half of the HT concentration), did not. Overall, the cool climate Hokkaido Zweigelt red wines produced vascular responses comparable to the existing body of research to date regarding the vasculoprotective effects from wines produced in warm climate regions across the world.

The data presented here have some limitations. The relatively small sample size is noted, but for a pilot study using a novel study design, it is reasonable. Interpersonal variability is another limitation, since substantial variability in postprandial responses to food consumption was also observed in the Personalised REsponses to DIetary Composition Trial (PREDICT) study that included 1,002 adult participants. The results of the PREDICT study showed highly variable postprandial responses in interleukin-6, glycoprotein acetylation, blood triglyceride, glucose, and insulin following a breakfast (86 g carbohydrate, 54 g fat, 16 g protein) and a lunch (71 g carbohydrate, 22 g fat, 10 g protein; consumed at the four-hour point) over six hours [75,76]. Individual profiles, including gut microbiome and genetic variants, greatly contributed to the variable postprandial outcomes [76]. Variations in personal genetic and gut microbiome profiles may influence personal ability to absorb and metabolize polyphenols such as hydroytyrosol [59,77], anthocyanins [78,79,80], and ellagitannins [81], and no gut microbiome profiles were assessed in the present study. The participants in this study were healthy adult males aged 50–70 years, and females or males of different age ranges, or those with elevated blood pressure or other vascular dysregulations were not assessed. The experimental design tested a single intake of red wine, and the results may not be generalizable to a longer duration of consumption. Sparkling white grape juice with added sugar was used as the control beverage in an attempt to mirror caloric and simple sugar content of the red wines. Although the sparkling white grape juice did not contain anthocyanins, other bioactive compounds such as gallic acid, tannins, and caftaric acid were present, albeit in very small amounts, which may have slightly impacted the outcome measures. Finally, inaccurate reporting of food intake is common in nutrition research [82] and the dietary changes noted in the second and third study visits may have influenced vascular function measures. However, the initial red wine group assignment was randomized, and when the treatment order was assessed as a factor that might influence the outcome measures, no evidence was found that the reported changes in diet were significant (Supplementary Tables S4 and S5).

## 5. Conclusions

A single intake of Hokkaido Zweigelt red wine produced in 2015 or 2018 resulted in a significant reduction in arterial stiffness in healthy adult males, while a sparkling white grape juice control beverage showed no effect. Consumption of the 2018 vintage significantly lowered SBP and DBP compared to the 2015 vintage or the control. Future studies with a larger sample size, detailing the red wine polyphenolic profiles including hydroxytyrosol and comparing different vintage years, are warranted.

## Figures and Tables

**Table 1:** Basic characteristics of the Hokkaido 2015 and 2018 Zweigelt red wines.

Item	Zweigelt 2015	Zweigelt 2018
Vineyard	Tsurunuma	Kitajima
Specific gravity	0.994	0.993
Alcohol (%)	12.34	12.54
Total acidity (g/L as tartaric acid)	5.23	5.73
pH	3.67	3.56
Total sulfur dioxide (SO_2_) (ppm)	98	149
Sugar (g/L)	5.6	3.5

Total SO_2_ is the sum of molecular, free, and bound SO_2_; ppm, part per million.

**Table 2:** Polyphenolic profile of a single serving of Hokkaido 2015 and 2018 Zweigelt red wine from the analyses conducted in 2019 and 2022 (prior to and at completion of the intervention, respectively) and white grape juice from the 2022 analysis.

Polyphenols (mg/240 mL)	Zweigelt 2015		Zweigelt 2018		White Grape Juice
	2019 Analysis	2022 Analysis	2019 Analysis	2022 Analysis	2022 Analysis
Gallic acid	9.12	8.64	4.80	5.52	0.17
Catechin	3.84	5.52	3.12	4.08	<0.05
Epicatechin	5.76	7.20	4.08	5.04	n.d.
Tannin	75.6	81.60	86.40	96.48	0.77
Caftaric acid	3.84	3.36	9.12	7.92	0.77
Caffeic acid	3.36	4.08	1.68	2.40	<0.05
Quercetin glycosides	2.64	2.64	2.64	1.68	<0.05
Quercetin	0.24	0.48	0.48	0.72	n.d.
Malvidin glucoside	4.80	0.72	31.92	2.64	n.d.
Polymeric anthocyanins	5.76	6.48	6.96	8.40	n.d.
Total anthocyanins	21.60	11.04	70.56	16.80	n.d.
Monomeric anthocyanins	15.84	4.56	63.60	8.40	n.d.
Resveratrol (cis + trans) (HPLC)	0.31	0.48	0.48	0.60	n.d.
Total polyphenol content (mg GAE)	455.28	674.00	NA	582.88	104.07

n.d.: not detected; NA: not available; HPLC: high-performance liquid chromatography; GAE: gallic acid equivalent.

**Table 3:** Tyrosol and hydroxytyrosol concentrations in the 2015 and 2018 Zweigelt red wines.

Wine Sample		Hydroxytyrosol (mg/L)	Tyrosol (mg/L)
**2015 Zweigelt**	Sample 1	8.81	84.57
	Sample 2	8.97	85.14
**Average (Mean ± SD)**		**8.89 ± 0.11**	**85.86 ± 0.40**
**2018 Zweigelt**	Sample 1	15.24	49.42
	Sample 2	15.31	49.63
**Average (Mean ± SD)**		**15.28 ± 0.05**	**49.53 ± 0.15**

**Table 4:** Baseline characteristics of participants.

**Demographics**	**Mean (SD), Range (min–max)**
Age (years)	58.6 (6.10), (51–69)
Weight (kg)	87.5 (13.85), (71–114)
Height (cm)	178.32 (6.84), (167–189.5)
BMI (kg/m^2^)	27.46 (4.02), (22.7–34.0)
Waist circumference (cm)	100.45 (14.02), (86–129)
**Selected CMP and CBC parameters**	**Mean (SD), reference range**
Glucose (mg/dL)	98.60 (10.81), (74–109)
Platelet count (K/MM^3^)	234.05 (87.60), (130–400)
**Vascular function parameters**	**Mean ± SEM**
RHI	2.25 ± 0.10
fRHI	0.78 ± 0.11
AI (% pulse pressure)	17.62 ± 6.46
AI75 (% pulse pressure)	7.70 ± 5.99
SBP (mmHg)	124.47 ± 2.70
DBP (mmHg)	82.20 ± 1.23
HR (bpm)	63.5 ± 2.68

RHI, reactive hyperemia index; fRHI, Framingham reactive hyperemia index; AI, augmentation index; AI75, augmentation index adjusted to 75 bpm; SBP, systolic blood pressure; DBP, diastolic blood pressure; HR, heart rate; bpm, beats per minute; SEM, standard error of mean; CMP, comprehensive metabolic panel; CBC, complete blood count.

**Table 5:** Changes in vascular outcomes from the baseline (T0) to two (T2) and four (T4) hours after each beverage consumption.

Outcomes (Change from Baseline)	Intervention Group						*p*-Value	
	Zweigelt 2015		Zweigelt 2018		White Grape Juice		Time	Treatment
	T2-T0	T4-T0	T2-T0	T4-T0	T2-T0	T4-T0		
	Mean ± SEM	Mean ± SEM	Mean ± SEM	Mean ± SEM	Mean ± SEM	Mean ± SEM		
AI	−16.32 ± 1.64	−9.64 ± 3.61	−18.27 ± 3.58 ^†^	−8.40 ± 3.85	−7.97 ± 2.67	−2.79 ± 5.30	0.001	0.002
AI75	−13.20 ± 1.37	−8.64 ± 3.29	−15.27 ± 3.82	−7.28 ± 3.74	−7.35 ± 2.65	−3.47 ± 5.46	0.01	0.04
SBP	0.13 ± 3.37	6.77 ± 3.08	−5.13 ± 3.67 ^†^	−3.13 ± 3.21	3.37 ± 2.33	2.33 ± 1.35	0.28	0.02
DBP	−3.63 ± 2.84	1.70 ± 1.83	−8.10 ± 1.60	−3.13 ± 1.75	−0.13 ± 1.12	−2.33 ± 1.39	0.07	0.02
HR	6.33 ± 2.06	1.50 ± 2.41	10.17 ± 1.67	3.57 ± 1.08	4.40 ± 1.64	1.57 ± 1.31	0.001	0.051
RHI	−0.04 ± 0.13	0.74 ± 0.24 ^‡^	0.09 ± 0.10	0.33 ± 0.21	0.07 ± 0.13	0.35 ± 0.14	0.003	0.62
fRHI	−0.08 ± 0.07	0.27 ± 0.10	−0.03 ± 0.06	0.14 ± 0.15	0.00 ± 0.07	0.18 ± 0.08	0.003	0.88
MaxA 1 µg collagen *	−0.45 ± 0.25	−0.10 ± 0.30	0.59 ± 0.33	−0.30 ± 0.34	0.35 ± 0.28	−0.10 ± 0.29	0.24	0.37
MaxA 3 µg collagen	−0.05 ± 0.13	−0.21 ± 0.08	0.23 ± 0.10	−0.10 ± 0.08	0.13 ± 0.11	−0.02 ± 0.14	0.02	0.17
MaxA 10 µM ADP *	0.08 ± 0.35	−0.04 ± 0.29	−0.11 ± 0.26	−0.53 ± 0.28	0.48 ± 0.34	−0.22 ± 0.22	0.12	0.36

* Data transformed by Johnson’s transformation to achieve normal distribution prior to the linear mixed model analysis. ^†^ Significantly different from control at the same time point, *p* < 0.05 upon post hoc analysis of treatment effect. ^‡^ Significantly different compared to T2-T0 value in the same group, *p* < 0.05 upon post hoc analysis of time effect. All data are reported as mean ± SEM; SEM, standard error of mean; RHI, reactive hyperemia index; fRHI, Framingham reactive hyperemia index; AI, augmentation index; AI75, augmentation index adjusted to 75 bpm; SBP, systolic blood pressure; DBP, diastolic blood pressure; HR, heart rate; bpm, beats per minute; MaxA, Maximal aggregation; ADP, adenosine diphosphate.

## Data Availability

Data are unavailable due to privacy or ethical restrictions.

## Supplementary Materials

The following supporting information can be downloaded at https://www.mdpi.com/article/10.3390/nu15184054/s1. Table S1: Reported baseline dietary intake assessed by a health habit questionnaire at the screening visit; Table S2: Changes in platelet aggregation assessed by Light Transmission Aggregometry (LTA) using 1 and 3 ug collagen and 10 mM ADP as the agonists from baseline to 2 h and from baseline to 4 h after beverage consumption; Table S3: Changes in platelet aggregation for each intervention group; Table S4: Participants’ 24 h recall dietary intake categorized by intervention; Table S5: Participants’ 24 h recall dietary intake categorized by study visit.
