# Supplementary material for: The Effect of Hokkaido Red Wines on Vascular Outcomes in Healthy Adult Men: A Pilot Study
Source: Nutrients. 2023 Sep 19;15(18):4054. doi: 10.3390/nu15184054 (PMC10535196; doi:10.3390/nu15184054)
Supplement: Supplementary file 1 [file nutrients-15-04054-s001.zip › nutrients-2590813-supplementary.pdf]

Table S1: Reported baseline dietary intake assessed by a health habit questionnaire

**Baseline dietary intake characteristics**	**Mean**	**SD**
Alcohol consumption (drinks/week)	4.6	2.8
Red wine (drinks/week)	2.8	1.5
White wine (drinks/week)	1.1	1.7
Total wine (drinks/week)	3.9	2.7
Beer (drinks/week)	1.6	1.5
Hard liquor (drinks/week)	1.2	1.6
Fruit intake (cups/week)	1.1	0.8
Vegetable intake (cups/week)	1.6	0.9
Tea intake (cups/day)	0.5	0.6
Coffee intake (cups/day)	0.9	0.7

Table S2: Changes in platelet aggregation assessed by Light Transmission Aggregometry (LTA) using 1 and 3 ug collagen and 10mM ADP as the agonists from baseline to 2 hr and from baseline to 4 hr after beverage consumption

Variable	Zweigelt 2015 (mean(SD))		Zweigelt 2018 (mean(SD))		White grape juice (mean(SD))		p-value (time effect)
	T2-T0	T4-T0	T2-T0	T4-T0	T2-T0	T4-T0	
10µM ADP MaxA	0.08(1.11)	-0.04(0.93)	-0.11(0.81)	-0.53(0.83)	0.48(1.07)	-0.22(0.70)	0.1264
10µM ADP Slope	-0.07(0.42)	-0.06(0.40)	0.01(0.30)	-0.07(0.22)	0.07(0.37)	0.10(0.39)	0.9400
10µM ADP AUC	0.08(1.12)	0.15(0.96)	-0.01(0.79)	-0.45(0.81)	0.49(1.08)	-0.08(0.82)	0.2443
1µg Collagen MaxA	-0.45(0.80)	-0.10(0.89)	0.59(1.05)	-0.30(1.09)	0.35(0.89)	-0.10(0.93)	0.2921
1µg Collagen Slope	-0.05(0.27)	0.01(0.31)	0.1(0.46)	-0.05(0.37)	0.04(0.36)	-0.14(0.30)	0.2111
1µg Collagen AUC	-0.14(0.33)	0.01(0.32)	0.16(0.40)	-0.08(0.34)	0.12(0.28)	-0.08(0.35)	0.2872
3µg Collagen MaxA	-0.05(0.40)	-0.21(0.24)	0.23(0.31)	-0.10(0.25)	0.13(0.33)	-0.02(0.45)	0.0378*
3µg Collagen Slope	0.25(0.31)	-0.01(0.24)	0.04(0.35)	-0.18(0.31)	0.02(0.42)	-0.13(0.36)	0.0241*
3µg Collagen AUC	0.08(0.36)	-0.06(0.21)	0.12(0.35)	-0.21(0.23)	0.19(0.32)	-0.12(0.43)	0.0038*

ADP, Adenosine diphosphate; MaxA, Maximal aggregation; AUC, area under the curve; Superscript * shows a significant difference between time points at $p<0.05$.

Table S3: Changes in platelet aggregation for each intervention group

**Data**	**Zweigelt 2015**		**Zweigelt 2018**		**White grape juice**		**p-value (treatment)**
	**Mean**	**SD**	**Mean**	**SD**	**Mean**	**SD**	
10μM ADP MaxA	0.0069	0.37386	-0.0765	0.30741	0.06581	0.32856	0.3582
10μM ADP Slope	-0.0641	0.39722	-0.0238	0.26324	0.08671	0.37021	0.4030
10μM ADP AUC	0.01369	0.38164	-0.0747	0.30233	0.05731	0.3445	0.3899
1μg Collagen MaxA	-0.0986	0.31214	0.04541	0.40565	0.04826	0.33046	0.3218
1μg Collagen Slope	-0.0199	0.27962	0.06755	0.42075	-0.0486	0.33326	0.5645
1μg Collagen AUC	-0.0667	0.32271	0.03877	0.38036	0.02462	0.32416	0.6250
3μg Collagen MaxA	-0.1249	0.337	0.06624	0.32289	0.0524	0.3915	0.2217
3μg Collagen Slope	0.12649	0.30305	-0.068	0.34266	-0.0522	0.38828	0.2169
3μg Collagen AUC	0.0123	0.2989	-0.0451	0.3376	0.03339	0.40441	0.7448

ADP, Adenosine diphosphate; MaxA, Maximal aggregation; AUC, area under the curve; Superscript * shows a significant difference between groups at $p<0.05$.

Table S4: Participants’ 24 hour recall dietary intake categorized by intervention

Variable	Zweigelt 2015		Zweigelt 2018		White grape juice		p-value
	Mean	SD	Mean	SD	Mean	SD	
Energy (kcal)	1483.86	539.45	1550.29	642.43	1800.55	832.06	NS
Protein (g)	74.99	48.76	75.72	40.01	93.29	63.41	NS
Total fat (g)	76.42	30.41	72.79	33.68	87.31	50.44	NS
Carbohydrate (g)	120.64	46.75	151.64	79.69	148.45	62.31	NS
Water (g)	1918.32	1289.45	2162.00	1361.15	2223.73	1461.73	NS
Alcohol (g)	3.44	10.89	0.00	0.00	8.71	19.18	NS
Caffeine (mg)	44.84	73.73	47.38	97.22	35.10	71.89	NS
Theobromine (mg)	2.73	7.83	0.36	1.14	21.49	51.43	NS
Total sugar (g)	33.62	32.23	49.12	42.86	53.76	34.95	NS
Total dietary fiber (g)	11.08	7.46	12.45	6.41	11.73	6.72	NS
Calcium (mg)	745.20	451.17	948.71	717.69	845.15	520.59	NS
Iron (mg)	8.55	2.40	11.30	5.52	10.90	5.18	NS
Sodium (mg)	3014.02	1788.79	3122.07	1275.25	3498.10	2282.26	NS
Total saturated fatty acids (g)	25.78	14.72	28.43	16.99	30.28	20.23	NS
Total monounsaturated fatty acids (g)	28.02	11.67	24.30	9.84	32.41	21.01	NS
Total polyunsaturated fatty acids (g)	15.65	5.66	14.34	6.37	17.19	11.83	NS

Table S5: Participants' 24 hour recall dietary intake categorized by study visit

Variable	Visit 1 (V1)		Visit 2 (V2)		Visit 3 (V3)		P-value (One-way ANOVA)
	Mean	SD	Mean	SD	Mean	SD	
Energy (kcal)	1953.39	575.73	1548.33	659.38	1332.98	686.49	NS
Protein (g)	94.56	58.57	81.24	51.42	68.20	42.15	NS
Total fat (g)	103.37	38.84	71.18	32.71	61.97	33.19	0.0337*
Carbohydrate (g)	158.55	49.97	143.41	70.85	118.76	68.20	NS
Water (g)	2117.74	1416.66	2025.47	1305.92	2160.85	1409.62	NS
Alcohol (g)	3.18	10.06	3.44	10.89	5.53	17.49	NS
Caffeine (mg)	44.38	98.19	30.49	61.34	52.45	80.45	NS
Theobromine (mg)	0.00	0.00	16.27	50.20	8.31	18.41	NS
Total sugar (g)	50.46	29.15	51.82	41.03	34.23	39.99	NS
Total dietary fiber (g)	12.72	5.75	12.21	8.17	10.34	6.30	NS
Calcium (mg)	912.95	416.12	747.32	518.42	878.80	744.14	NS
Iron (mg)	13.54	4.61	9.24	3.81	7.98	3.65	0.0124*
Sodium (mg)	4018.21	1902.05	3265.74	1867.17	2350.25	1234.34	NS
Total folate (ug)	358.83	151.42	250.56	124.23	209.62	110.06	0.0434*
Folate (ug_DFE)	461.07	227.55	305.40	159.91	256.19	145.59	NS
Total saturated fatty acids (g)	39.70	17.93	24.24	12.35	20.56	14.94	0.0216*
Total monounsaturated fatty acids (g)	36.43	17.20	26.48	13.52	21.83	10.42	NS
Solid fats (g)	54.67	28.11	29.24	17.97	25.19	22.54	0.0434*

Superscript * represents a significant difference at V1 compared to V2 and V3 at $p<0.05$. Solid fats are fats that are naturally present in animal products or hydrogenated/partially hydrogenated vegetable oils including lard, tallow, butter, shortening, palm kernel, coconut oils, cocoa butter, margarines; DFE, dietary folate equivalents
